# Reducing-End Functionalization of 2,5-Anhydro-d-mannofuranose-Linked Chitooligosaccharides by Dioxyamine: Synthesis and Characterization

**DOI:** 10.3390/molecules25051143

**Published:** 2020-03-04

**Authors:** Maxence Coudurier, Jimmy Faivre, Agnès Crépet, Catherine Ladavière, Thierry Delair, Christophe Schatz, Stéphane Trombotto

**Affiliations:** 1 Ingénierie des Matériaux Polymères, IMP UMR CNRS 5223, Univ Lyon, Université Claude Bernard Lyon 1, F-69622 Villeurbanne, France; maxence.coudurier@ens-lyon.fr (M.C.); faivrejimmy@gmail.com (J.F.); agnes.crepet@univ-lyon1.fr (A.C.); catherine.ladaviere@univ-lyon1.fr (C.L.); thierry.delair@univ-lyon1.fr (T.D.); 2 Laboratoire de Chimie des Polymères Organiques (LCPO), Univ Bordeaux, CNRS, Bordeaux INP, UMR 5629, F-33600 Pessac, France; schatz@enscbp.fr

**Keywords:** chitosan, oligomer, reducing-end, conjugation, building-block, oxyamine

## Abstract

The nitrous acid depolymerization of chitosan enables the synthesis of singular chitosan oligosaccharides (COS) since their reducing-end unit is composed of 2,5-anhydro-d-mannofuranose (amf). In the present study, we describe a chemical method for the reducing-end conjugation of COS-amf by the commercially available dioxyamine *O,O′*-1,3-propanediylbishydroxylamine in high mass yields. The chemical structure of resulting dioxyamine-linked COS-amf synthesized by both oximation and reductive amination ways were fully characterized by ^1^H- and ^13^C-NMR spectroscopies and MALDI-TOF mass spectrometry. The coupling of chemically attractive linkers such as dioxyamines at the reducing end of COS-amf forms a relevant strategy for the development of advanced functional COS-based conjugates.

## 1. Introduction

**Scheme 1:**
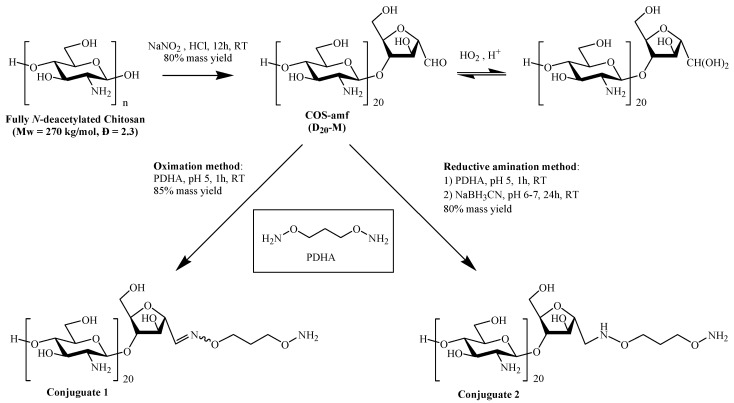
Synthesis of conjugates **1** and **2** from COS-amf (D_20_-M) prepared by nitrous acid depolymerization of fully *N*-deacetylated chitosan.

Chitosan is a linear copolymer of β-(1→4)-linked d-glucosamine (GlcN or D) and *N*-acetyl d-glucosamine (GlcNAc or A) units in various proportions. Although present in biomass, chitosan is generally obtained by chemical or enzymatic *N*-deacetylation of chitin, the second most abundant naturally occurring polymer, produced industrially from shells of crustaceans and squid pens [1,2,3,4]. Chitosan oligomers, also named chitooligosaccharides (COS), have recently received considerable attention as functional biomacromolecules with a wide range of potential applications in food, agriculture, medicine, pharmaceutics, and cosmetics. COS take advantage of various interesting physico–chemical and biological properties, including principally water-solubility, biodegradability, biocompatibility, antibacterial, antiviral, and antifungal activities [5,6,7,8,9]. In order to develop new COS-based materials with advanced and significant added-value applications, the functionalization of COS with chemically reactive groups is currently being explored intensively [9,10,11,12,13,14,15]. In this context, we have recently described a powerful and versatile method for the introduction of “clickable” chemical groups such as alkene, alkyne, azide, hydrazide, and thiol at the reducing end of COS synthesized by nitrous acid depolymerization of chitosan [16]. The chemical structure of these COS is indeed particularly interesting because of the presence of the 2,5-anhydro-d-mannofuranose (amf or M) unit at the reducing end (Scheme 1). Hence, the amf unit does not mutarotate in aqueous solutions unlike GlcNAc and GlcN units. That means its aldehyde group does not participate in the hemiacetal-aldehyde equilibrium and is certainly more available for chemical reactions [17]. Moreover, as amine groups of GlcN units of COS-amf are not involved in the functionalization, this method has the advantage of not modifying the chemical nature of the COS backbone and consequently to preserve their intrinsic physico–chemical and biological properties.

Thanks to their high nucleophilicity in a large range of pH, oxyamines (pKa ~4-5 [18]) have recently been described as a powerful chemo-selective tool for the reducing-end conjugation of various oligosaccharides in the development of new glycoconjugates [19,20]. Thus, dioxyamines conjugated to oligosaccharides could be particularly relevant precursors for the elaboration of advanced oligosaccharides-based materials. In this context, Vilkøren Mo et al. [21] have recently described a kinetic and structural study of the conjugation of the dioxyamine *O,O′*-1,3-propanediylbishydroxylamine (PDHA) to enzymatically produced COS having *N*-acetyl d-glucosamine at the reducing end. To our knowledge, this reducing-end conjugation approach has never been investigated in the case of COS with amf residue at the reducing end. In the present study, we therefore describe the reducing-end conjugation of COS-amf by PDHA in terms of reaction conditions and mass yields (Scheme 1). The main objective of this study was to develop an original chemical method for the preparation of new COS-based building blocks functionalized at their reducing end by an oxyamine group. The full structural characterization of these dioxyamine-linked COS conjugates by Nuclear Magnetic Resonance (NMR) spectroscopy and Matrix Assisted Laser Desorption Ionization - Time of Flight (MALDI-TOF) mass spectrometry (MS) are also discussed in detail.

## 2. Materials and Methods

### 2.1. Materials

Commercial chitosan (batch 244/020208; degree of *N*-acetylation: DA < 1%, Mw = 270 kg/mol; Mn = 115 kg/mol, dispersity Ð = 2.3) was supplied by Mahtani Chitosan Ltd. (Veraval, India). Sodium nitrite (NaNO_2_, purity > 99%), *O,O′*-1,3-propanediylbishydroxylamine dihydrochloride (PDHA, purity > 98%), and sodium cyanoborohydride (NaBH_3_CN, purity > 95%) were provided by Sigma–Aldrich (Saint-Quentin Fallavier, France). All other chemicals and solvents were obtained from commercial sources and were of analytical grade.

### 2.2. Analytical Methods

**NMR spectroscopy**. ^1^H and ^13^C NMR spectra were recorded on a Bruker 500 MHz spectrometer at 300 K. All samples were dissolved at 10 mg/mL in D_2_O with 5 μL HCl 12 N and were transferred to 5 mm NMR tubes. Trimethylsilyl-3-propionic-2,2,3,3-D_4_ acid sodium salt (99% atom D, TMSPA from Sigma–Aldrich, Saint-Quentin Fallavier, France) was used as internal reference (δ 0.00 and −2.25 ppm for ^1^H and ^13^C NMR, respectively). Proton and carbon signals were assigned thanks to 2D NMR techniques (i.e., COSY and HSQC).

**MALDI-TOF mass spectrometry**. MALDI-TOF mass spectra were acquired with a Voyager-DE STR (AB Sciex, Framingham, MA, USA) equipped with a nitrogen laser emitting at 337 nm with a 3 ns pulse. The instrument was operated in the linear or reflectron mode. Ions were accelerated to a final potential of 20 kV. The positive ions were detected in all cases. Mass spectra were the sum of 300 shots and an external mass calibration of mass analyzer was used (mixture of peptides from SequazymeTM standards kit, AB Sciex). The matrix used for all experiments was 2,5-dihydroxybenzoic acid (DHB) purchased from Sigma–Aldrich and used directly without further purification. The solid matrix and samples were dissolved at 10 mg/mL in water. A volume of 9 µL matrix solution was then mixed with 1 µL of sample solution. An aliquot of 1 µL of each resulting solution was spotted onto the MALDI sample plate and air-dried at room temperature.

### 2.3. Synthesis Procedures and Structural Characterizations

**COS-amf (D_20_-M)**. Chitosan (2.1g, 13 mmol of GlcN unit) was solubilized in 100 mL of deionized water by addition of 1.2 mL HCl (37% *w*/*w*). A freshly prepared 5 mL aqueous solution of NaNO_2_ (90 mg, 0.13 mmol for GlcN/NaNO_2_ molar ratio = 10:1) was added and the reaction was allowed to proceed for 12 h at room temperature. Oligomers were precipitated by addition of ammonium hydroxide solution (28% *w*/*w*) to pH ~8–9, washed several times with deionized water until neutral pH, then freeze-dried leading to COS-amf (1.7 g, 80% mass yield) as a white powder. MALDI-TOF MS (positive reflectron mode): major peak at *m*/*z* 1473.9 assigned to H-(C_6_H_11_O_4_N)_8_-C_6_H_9_O_5_ (*m*/*z* monoisotopic calcd for [C_54_H_98_O_37_N_8_Na]^+^ = 1473.6 *m*/*z*). ^1^H-NMR (500 MHz, D_2_O): δ (ppm) 5.10 (d, *J* = 5.4 Hz, 1H, H-1 amf gem diol), 4.90–4.70 (m, 20H, H-1 GlcN), 4.45 (t, *J* = 4.9 Hz, 1H, H-3 amf), 4.23 (t, *J* = 4.9 Hz, 1H, H-4 amf), 4.13 (m, 1H, H-5 amf), 4.05–3.45 (m, 103H, H-2 and H-6 amf, H-3 to H-6 GlcN), 3.20–3.10 (m, 20H, H-2 GlcN). ^13^C-NMR (125 MHz, D_2_O): δ (ppm) 99.2 (C-1# GlcN), 98.6 (C-1 GlcN), 89.8 (C-1 amf gem diol), 86.5 (C-4 amf), 85.6 (C-2 amf), 82.6 (C-5 amf), 77.2 (C-3 amf), 77.0 (C-4 GlcN), 76.9 (C-5* GlcN), 75.3 (C-5 GlcN), 72.5 (C-3* GlcN), 71.0 (C-3 GlcN), 70.2 (C-4* GlcN), 61.4 (C-6 amf), 60.9 (C-6* GlcN), 60.6 (C-6 GlcN), 56.5 (C-2 GlcN), and 56.2 (C-2* GlcN). Note that * denotes carbon atoms of the non-reducing end GlcN unit and # for carbon atoms of the GlcN unit linked to the amf unit.

**Conjugate 1**. COS-amf (0.5 g, 0.15 mmol of amf unit) was dissolved into 10 mL of deionized water (pH 5). 270 mg of PDHA (10 eq./amf unit, 1.5 mmol) were dissolved into 10 mL of deionized water. The COS-amf solution was added dropwise to the PDHA solution. The pH of the solution was then adjusted to 5 by addition of Na_2_CO_3_, and the mixture was stirred for 1 h at room temperature. At the end of the reaction, oligomers were precipitated by addition of ammonium hydroxide solution (28% w/w) to pH ~8–9, washed several times with deionized water until neutral pH, then freeze-dried leading to the conjugate **1** (0.42 g, 85% mass yield) as white powder. MALDI-TOF MS (positive reflectron mode): major peak at 1883.8 *m*/*z* assigned to H-(C_6_H_11_O_4_N)_10_-C_9_H_17_N_2_O_6_ (*m*/*z* monoisotopic calcd for [C_69_H_128_O_46_N_12_Na]^+^ = 1883.8 *m*/*z*). ^1^H-NMR (500 MHz, D_2_O): δ (ppm) 7.59 (d, *J* 5.9Hz, 0.8H, CH=N of *E*-isomer), 7.00 (d, *J* 5.0Hz, 0.2H, CH=N of *Z*-isomer), 5.04 (m, 0.2H, H-2 amf of *Z*-isomer), 4.90 (m, 20H, H-1 GlcN), 4.60–4.40 (m, 1.8H, H-2 amf of *E*-isomer, H-3 amf), 4.30–4.10 (m, 4H, H-4 amf, H-5 amf, CH_2_ONH_2_), 4.05–3.40 (m, 104H, H-3 to H-6 GlcN, H-6 amf, =NOCH_2_), 3.25–3.05 (m, 20H, H-2 GlcN), 2.10–1.90 (m, 2H, CH_2_CH_2_ONH_2_). ^13^C-NMR (125 MHz, D_2_O): δ (ppm) 151.2 (CH=N, *Z*-isomer), 150.2 (CH=N, *E*-isomer), 99.6 (C-1# GlcN), 99.2 (C-1 GlcN), 85.8 (C-4 amf), 83.1 (C-5 amf), 80.6 (C-2 amf of *E*-isomer), 79.7 (C-2 amf of *Z*-isomer), 78.3 (C-3 amf), 77.1 (C-4 GlcN), 76.8 (C-5* GlcN), 75.2 (C-5 GlcN), 73.0 (=NOCH_2_), 72.8 (C-3* GlcN), 71.5 (C-3 GlcN), 71.3 (CH_2_ONH_2_), 70.0 (C-4* GlcN), 61.6 (C-6 amf), 60.8 (C-6* GlcN), 60.4 (C-6 GlcN), 56.4 (C-2 GlcN), 56.1 (C-2* GlcN), and 27.6 (CH_2_CH_2_ONH_2_). Note that * denotes carbon atoms of the non-reducing end GlcN unit and # for carbon atoms of the GlcN unit linked to the amf unit.

**Conjugate 2**. COS-amf (0.5 g, 0.15 mmol of amf unit) was dissolved into 10 mL of deionized water (pH 5). PDHA (270 mg; 10 eq./amf unit, 1.5 mmol) was dissolved into 10 mL of deionized water. The COS-amf solution was added dropwise to the PDHA solution. The pH of the solution was then adjusted to 5 by addition of Na_2_CO_3_. After the mixture was stirred at room temperature for 1 h, sodium cyanoborohydride (50 eq./amf unit) was added portionwise to the mixture. The solution was then stirred for 24 h at room temperature. At the end of the reaction, oligomers were precipitated by addition of ammonium hydroxide solution (28% w/w) to pH ~8–9, washed several times with deionized water until neutral pH, then dialyzed (cellulose acetate membrane, MWCO 1kDa, Spectrum Labs) against deionized water, and finally freeze-dried leading to the conjugate **2** (0.4 g, 80% mass yield) as white powder. MALDI-TOF MS (positive reflectron mode): major peak at 1402.8 *m*/*z* assigned to H-(C_6_H_11_O_4_N)_7_-C_9_H_19_N_2_O_6_ (*m*/*z* monoisotopic calcd for [C_51_H_97_O_34_N_9_Na]^+^ = 1402.6 *m*/*z*). ^1^H-NMR (500 MHz, D_2_O): δ (ppm) 4.90 (m, 20H, H-1 GlcN), 4.40–4.10 (m, 6H, H-2 to H-5 amf, CH_2_ONH_2_), 4.05–3.40 (m, 104H, H-3 to H-6 GlcN, H-6 amf, NHOCH_2_), 3.30–3.05 (m, 22H, H-2 GlcN, CH_2_NHO), 2.10-1.90 (m, 2H, CH_2_CH_2_ONH_2_). ^13^C-NMR (125 MHz, D_2_O): δ (ppm) 98.3 (C-1# GlcN), 98.1 (C-1 GlcN), 86.7 (C-4 amf), 82.3 (C-5 amf), 79.9 (C-2 amf), 78.4 (C-3 amf), 77.0 (C-5* GlcN), 76.9 (C-4 GlcN), 75.3 (C-5 GlcN), 72.9 (CH_2_ONH_2_), 72.3 (C-3* GlcN), 70.6 (C-3 GlcN), 70.4 (NHOCH_2_)_,_ 70.2 (C-4* GlcN), 61.8 (C-6 amf), 60.9 (C-6* GlcN), 60.5 (C-6 GlcN), 56.4 (C-2 GlcN), 56.1 (C-2* GlcN), 52.6 (CH_2_NO), and 27.1 (CH_2_CH_2_ONH_2_). Note that * denotes carbon atoms of the non-reducing end GlcN unit and # for carbon atoms of the GlcN unit linked to the amf unit.

## 3. Results and Discussion

The COS-amf sample used in this study was produced by nitrous acid depolymerization of a fully *N*-deacetylated chitosan (Scheme 1). The chemical structure of COS-amf is of the type D_n_-M, where *n* is the average number of contiguous uninterrupted GlcN units equal to 20. This sample was prepared according to the chemical method that Moussa et al. [16] have previously described in detail. Briefly, the nitrous acid depolymerization was typically performed by mixing a dilute aqueous acid solution of the fully *N*-deacetylated chitosan (2% w/w) and a stoichiometric quantity of NaNO_2_ at room temperature for 12 h. At the end of the reaction, COS-amf was isolated by precipitation in a basic medium. Then, the precipitate was abundantly washed with deionized water in order to remove soluble impurities and finally dried by lyophilisation. Thus, COS-amf was produced in a gram-scale quantity with a mass yield of 80% after purification. The expected chemical structure of COS-amf was fully confirmed by both ^1^H and ^13^C NMR and MALDI-TOF MS analyses (Figures S3–S9 in Supplementary Materials). ^1^H and ^13^C NMR spectra were in good agreement with those given by Moussa et al. [16] for analogous COS-amf with degrees of polymerization ranging from 10–45. However, note that assignments of some signals in ^1^H and ^13^C NMR spectra of COS-amf were erroneously described in [16] and were here corrected (Figures S4–S9 in Supplementary Materials). The average number of GlcN units was determined by ^1^H NMR, by comparing the peak intensities of two signals at 4.45 ppm and 3.15 ppm (Figure S4 in Supplementary Materials), corresponding to the H-3 proton of the amf unit and the H-2 proton of GlcN units, respectively, as described in [16].

**Figure 1:**
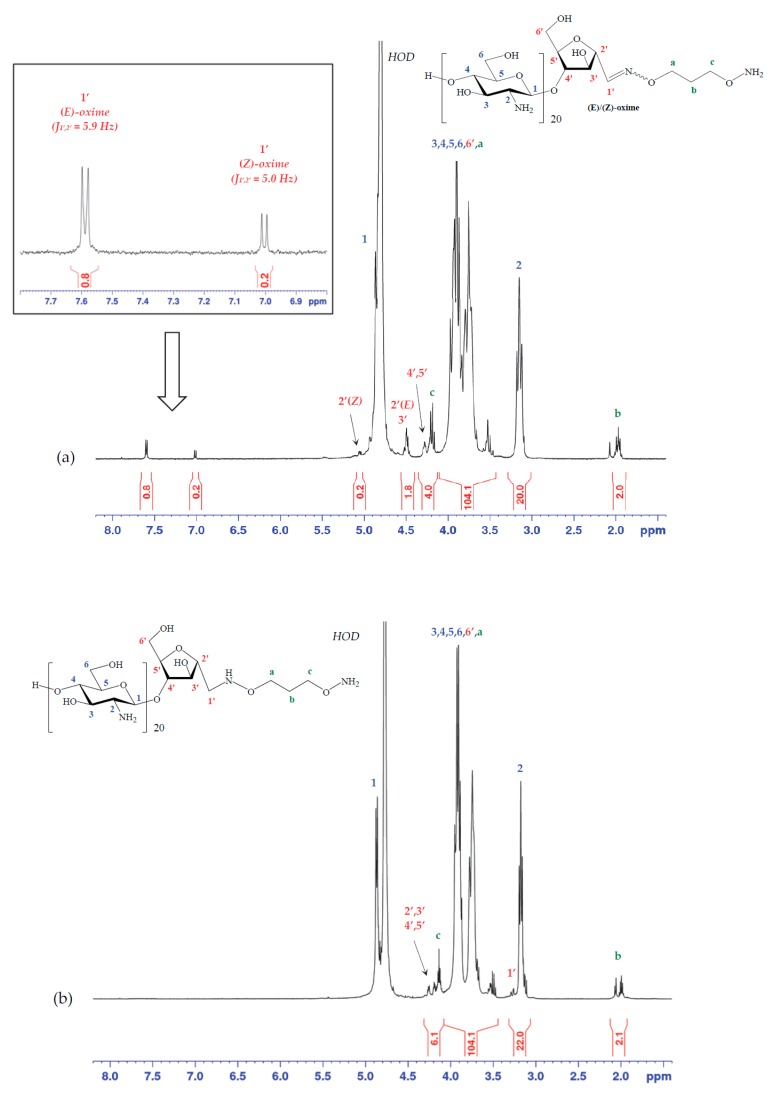
^1^H NMR spectra (D_2_O, 500 MHz, RT) of conjugates **1** (**a**) and **2** (**b**).

The conjugation of the dioxyamine PDHA to COS-amf was investigated by both oximation and reductive amination approaches, thereby enabling the elaboration of COS-based conjugates with cleavable and non-cleavable linkers, respectively. Concerning the oximation conjugation, the reaction between COS-amf and PDHA was carried out with a large excess of the dioxyamine (10 molar eq./amf unit) in order to promote the coupling reaction with one oxyamine group only. Based on results of Baudendistel et al. [20], the pH of the solution was kept constant at pH 5 during the reaction, firstly to solubilize COS-amf in the aqueous solvent, but also to ensure that the amine group of PDHA (pKa 4.2 [21]) is more nucleophilic than amine groups of COS-amf (pKa ~6.2–7 [14]) towards the aldehyde group of the amf unit. In this condition of pH, the kinetic study of the reaction by ^1^H NMR showed clearly: (i) the formation of the oxime group (CH=NO) characterized by two signals at 7.59 and 7.00 ppm for (*E*)- and (*Z*)-oxime protons, respectively [21] (Figure 1a); and (ii) the disappearance of the doublet signal at 5.10 ppm corresponding to the amf aldehyde group in its hydrated form (–CH(OH)_2_, below named gem diol group [16,17]; Figure S4 in Supplementary Materials). Thus, after only 30 min of reaction at RT, the total conversion of the gem diol group into oxime was observed. It is worth noting that when the oximation reaction was performed at pH 4, the gem diol conversion was slower than at pH 5. Moreover, the occurrence of numerous new peaks between 8.2–5.5 ppm in the ^1^H NMR spectrum was observed (Figure S10 in Supplementary Materials), indicating likely secondary reactions and/or the instability of the conjugate **1** in such acidic conditions at Room temperature (RT). Consequently, after 1 h of reaction at pH 5, the conjugate **1** was isolated easily from the reaction mixture by precipitation in alkaline conditions, after increasing the pH by addition of concentrated ammonia. A further purification of the precipitate by washing with deionised water led to conjugate **1** in 85% mass yield after lyophilisation. Thanks to homo- and heteronuclear correlation NMR analyses (i.e., Correlated Spectroscopy (COSY) and Heteronuclear Single Quantum Coherence (HSQC) techniques), ^1^H and ^13^C spectra of conjugate **1** were fully assigned in accordance with the oximation coupling between COS-amf and PDHA (Figures S12–S15 in Supplementary Materials). In particular, the ^1^H NMR spectrum of conjugate **1** (Figure 1a) showed clearly the presence of both (*E*)- and (*Z*)-oxime protons (ratio *E*/*Z* 80:20) at 7.59 ppm and 7.00 ppm, respectively (as stated above), in addition to the presence of CH_2_ protons of CH_2_ONH_2_ and =NOCH_2_ groups at 4.15 ppm and 3.85 ppm, respectively (Figure S14 in Supplementary Materials).

Moreover, the ^13^C NMR spectrum of the conjugate **1** showed two signals at 151.2 ppm and 150.2 ppm for the CH=NO carbon ((*Z*)- and (*E*)-oxime, respectively) and two signals at 73.0 ppm and 71.3 ppm for =NOCH_2_ and CH_2_ONH_2_ carbons, respectively (Figures S12 and S15 in Supplementary Materials). Finally, MALDI-TOF MS analyses was also useful to confirm the expected chemical structure of conjugate **1**. Indeed, the mass spectrum (Figure S11 in Supplementary Materials) showed clearly a distribution of peaks ranging from ca. 1240–3817 *m*/*z*, with a mass difference of 161 *m*/*z* between two consecutive peaks corresponding to the molar mass of the GlcN unit. Each peak mass was in complete agreement with the molar mass calculated from the general conjugate **1** sodium adduct formula [H-(C_6_H_11_O_4_N)_x_-C_9_H_17_N_2_O_5,_ Na]^+^, where x corresponds to the exact GlcN unit number varying from 6–22 in the mass spectrum.

As oxime conjugates can be labile to acid hydrolysis [22], we also investigated in a complementary manner the non-cleavable conjugation of COS-amf with PDHA by reductive amination. Thus, the reductive amination was carried out in conditions of pH, temperature, and molar equivalents of reagents similar to the oximation conjugation as mentioned above. Typically, COS-amf and PDHA (10 molar eq./amf unit) were solubilized in deionized water at pH 5. The mixture was stirred at room temperature for 1 h, and then NaBH_3_CN was added to the solution in large excess (50 molar eq./amf unit) so that the reduction of the oxime group was complete. As the toxicity of NaBH_3_CN may raise health, safety, and environment concerns, the use of less hazardous reducing agents such as α-picoline borane could be an alternative to NaBH_3_CN [21]. After stirring at RT for 24 h, the addition of concentrated ammonia (28% w/w) resulted in the precipitation of conjugate **2** in 80% mass yield after purification (Scheme 1). The chemical structure of conjugate **2** was analyzed by both NMR and MALDI-TOF MS. Thus, ^1^H and ^13^C NMR spectra were fully assigned thanks to COSY and HSQC NMR analyses (Figures S19 and S20 in Supplementary Materials), showing the complete reduction of the oxime link between COS-amf and PDHA.

In particular, the comparison of NMR spectra of conjugates **1** and **2** showed the disappearance of the oxime signals at 7.59 ppm and 7.00 ppm in the ^1^H NMR spectrum (Figure 1), and the presence of the resultant CH_2_NO group at 3.30–3.10 ppm and 52.6 ppm in ^1^H and ^13^C NMR spectra, respectively (Figures S19 and S20 in Supplementary Materials). Moreover, in the ^1^H NMR spectrum of conjugate **2** (Figure 1b), the integration values of signals from COS-amf and PDHA residues (ratio COS-amf/PDHA 20:1) were in good agreement with a single coupling between COS-amf and PDHA, which was also confirmed by MALDI-TOF MS analyses (Figure S16 in Supplementary Materials).

Finally, the success of the synthesis of these two COS-based building blocks functionalized at their reducing end by an oxyamine group opens the way for the development of advanced functional COS-based conjugates, such as diblock copolymers [16].

## Supplementary Materials

The following are available online. NMR spectra, MALDI-TOF mass spectra and size-exclusion chromatograms are available online.
