# Supplementary material for: Reducing-End Functionalization of 2,5-Anhydro-d-mannofuranose-Linked Chitooligosaccharides by Dioxyamine: Synthesis and Characterization
Source: Molecules. 2020 Mar 4;25(5):1143. doi: 10.3390/molecules25051143 (PMC7179158; doi:10.3390/molecules25051143)
Supplement: Supplementary file 1 [file molecules-25-01143-s001.pdf]

Supplementary Materials

Reducing-end Functionalization of 2,5-Anhydro-D-mannofuranose-linked Chitooligosaccharides by Dioxyamine: Synthesis and Characterization

Maxence Coudurier[1], Jimmy Faivre[1], Agnès Crépet[1], Catherine Ladavière[1], Thierry Delair[1], Christophe Schatz[2], Stéphane Trombotto[1*]

[1] Ingénierie des Matériaux Polymères, IMP UMR CNRS 5223, Univ Lyon, Université Claude Bernard Lyon 1, F-69622, Villeurbanne, France
[2] Laboratoire de Chimie des Polymères Organiques (LCPO), Univ Bordeaux, CNRS, Bordeaux INP, UMR 5629, F-33600, Pessac, France
* Correspondence. stephane.trombotto@univ-lyon1.fr; Tel.: +33-472-431-210

List of Supplementary Materials

Table S1. Chemical structures and chemical formulas of COS-amf, conjugates **1** and **2** 3

Figure S1. ^{1}H-NMR spectrum (D_2O, 500 MHz, 298 K) of commercial chitosan 4

Figure S2. Size-exclusion chromatogram report of commercial chitosan 5

Figure S3. MALDI-TOF mass spectrum (positive linear mode) of COS-amf (D_{20}-M) 6

Figure S4. ^{1}H-NMR spectrum (D_2O, 500 MHz, 298 K) of COS-amf (D_{20}-M) 6

Figure S5. ^{13}C-NMR spectrum (D_2O, 125 MHz, 298 K) of COS-amf (D_{20}-M) 7

Figure S6. DEPT 135 ^{13}C-NMR spectrum (D_2O, 125 MHz, 298 K) of COS-amf (D_{20}-M) 7

Figure S7. 2D COSY-NMR spectrum (D_2O, 500 MHz, 298 K) of COS-amf (D_{20}-M) 8

Figure S8. 2D HSQC-NMR spectrum (D_2O, 500 MHz, 298 K) of COS-amf (D_{20}-M) 9

Figure S9. 2D HMBC-NMR spectrum (D_2O, 500 MHz, 298 K) of COS-amf (D_{20}-M) 10

Figure S10. NMR spectra (region from 5.3 to 8.2 ppm) of the oximation of COS-amf (D_{20}-M) with PDHA at pH 4 and pH 5 after 1h of reaction 11

Figure S11. MALDI-TOF mass spectrum (positive reflectron mode) of conjugate **1** 11

Figure S12. ^{13}C-NMR spectrum (D_2O, 125 MHz, 298 K) of conjugate **1** 12

Figure S13. DEPT 135 ^{13}C-NMR spectrum (D_2O, 125 MHz, 298 K) of conjugate **1** 12

Figure S14. 2D COSY-NMR spectrum (D_2O, 500 MHz, 298 K) of conjugate **1** 13

Figure S15. 2D HSQC-NMR spectrum (D_2O, 500 MHz, 298 K) of conjugate **1** 13

Figure S16. ^{1}H DOSY NMR spectrum (D2O, 500 MHz, 298 K) of conjugate **1** 14

Figure S17. MALDI-TOF mass spectrum (positive reflectron mode) of conjugate **2** 14

Figure S18. ^{13}C-NMR spectrum (D_2O, 125 MHz, 298 K) of conjugate **2** 15

Figure S19. DEPT 135 ^{13}C-NMR spectrum (D_2O, 125 MHz, 298 K) of conjugate **2** 15

Figure S20. 2D COSY-NMR spectrum (D_2O, 500 MHz, 298 K) of conjugate **2** 16

Figure S21. 2D HSQC-NMR spectrum (D_2O, 500 MHz, 298 K) of conjugate **2** 16

Figure S22. ^{1}H DOSY NMR spectrum (D2O, 500 MHz, 298 K) of conjugate **2** 17

Table S1. Chemical structures and chemical formulas of COS-amf, conjugates **1** and **2**

Compound	Chemical Structure	Chemical Formula
**COS-amf (D_{20}-M)**	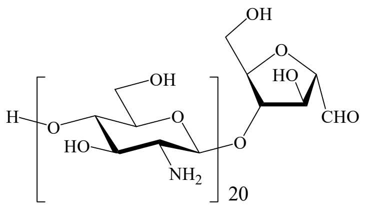	H-$(C_6H_{11}O_4N)_{20}$-$C_6H_9O_5$
**Conjugate 1**	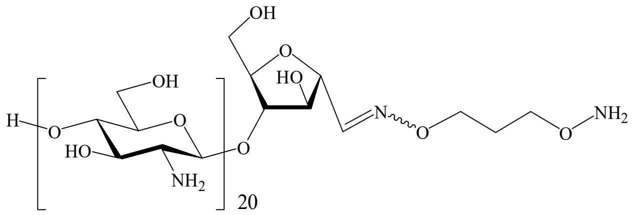	H-$(C_6H_{11}O_4N)_{20}$-$C_9H_{17}N_2O_6$
**Conjugate 2**	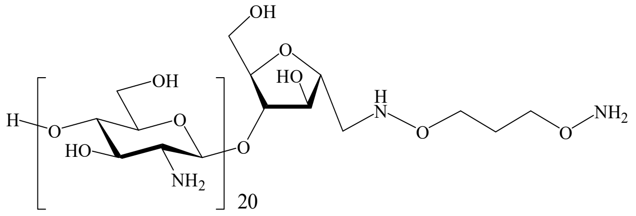	H-$(C_6H_{11}O_4N)_{20}$-$C_9H_{19}N_2O_6$

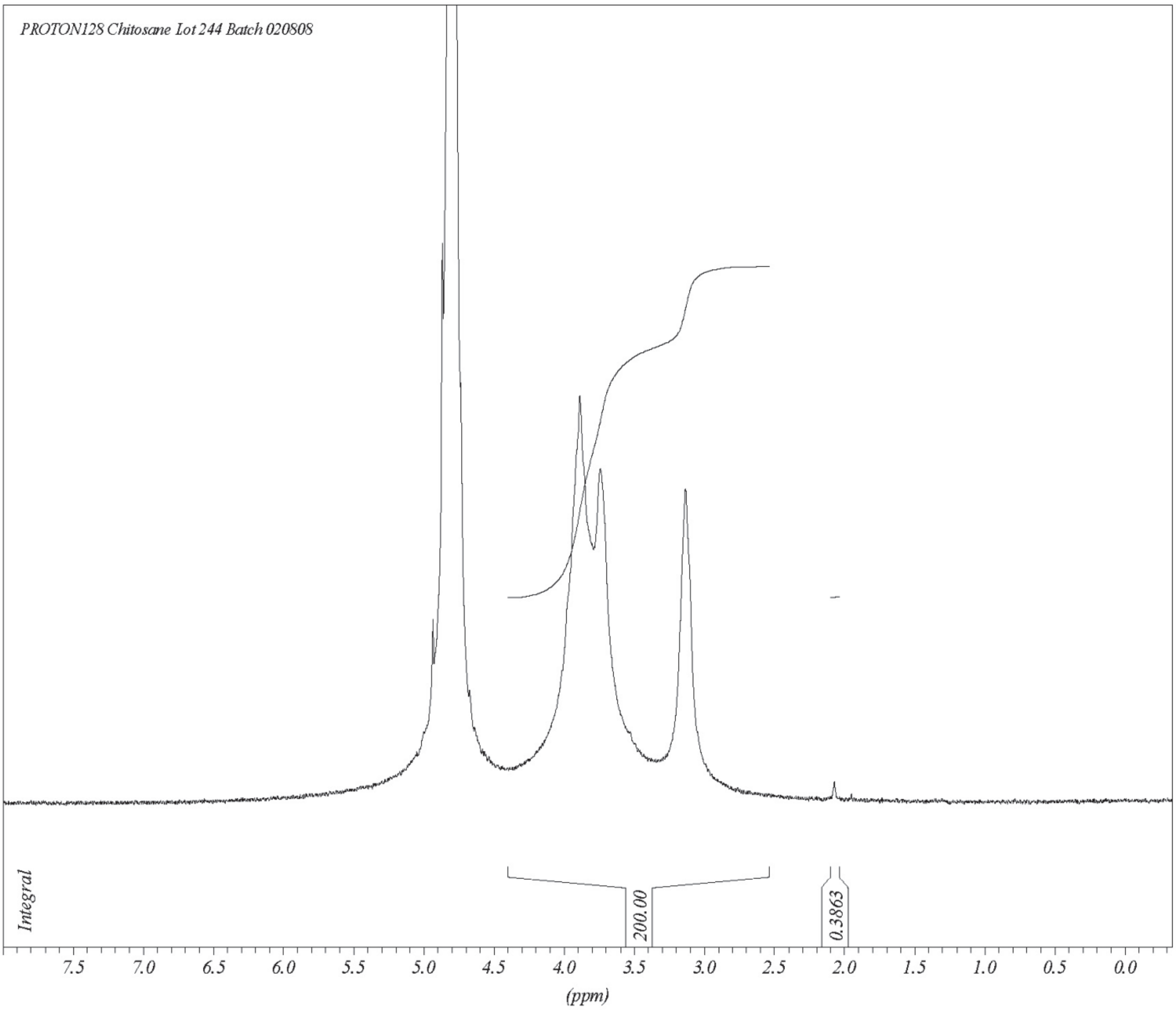

Figure S1. ^{1}H-NMR spectrum (D_2O, 500 MHz, 298 K) of commercial chitosan

(Batch 244/020208; degree of *N*-acetylation. DA < 1%, Mw = 270 kg/mol; Mn = 115 kg/mol, dispersity **Đ** = 2.3)

File Name: F:244-2[10oct2012].afe6
Collection Operator: LMPB-AR2000\Aqueux (LMPB-AR2000\Aqueux (Aqueux))
Processing Operator: UNIV-LYON1\stephane.trombotto (TROMBOTTO STEPHANE)

Sample: 244-2
Concentration: 1.010 mg/mL
Injected Volume: 100.0 µL

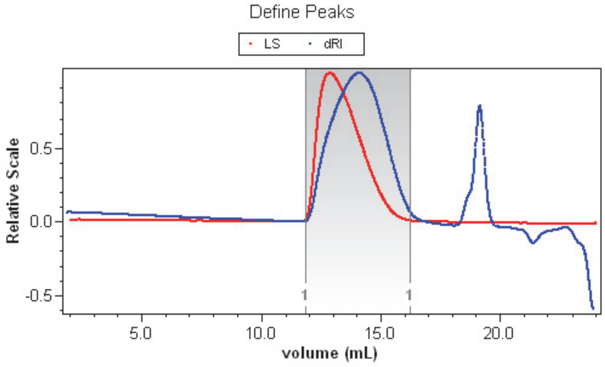

Configuration

Notes:
Colonnes : TSK6000 et TSK2500, Solvant filtré sur CME 0,1 et échantillon filtré sur CME 0,45

Concentration Source: RI
Flow Rate: 0.500 mL/min

Light Scattering Instrument: DAWN EOS
Cell Type: K5
Wavelength: 690.0 nm
Calibration Constant: 7.4800×10^{-6} 1/(V cm)

RI Instrument: Optilab rEX

Solvent: Tampon AcAc/AcNH pH 4.5
Refractive Index: 1.330

Processing

Collection Time: Thursday October 11, 2012 03:35:55 AM Paris, Madrid (heure d'été)
Processing time: Thursday October 11, 2012 11:05:24.828 AM Paris, Madrid (heure d'été)

Peak settings:

Peak Name	Peak 1
Light Scattering Model	Zimm
Fit Degree	1
dn/dc (mL/g)	0.1980
A2 (mol mL/g^2)	0.000

Results Fitting Procedure:
Data Fit Model Degree R^2 Extrapolation

Results

Peak Results

	Peak 1
Masses	
Injected Mass (µg)	101.00
Calculated Mass (µg)	77.56
Molar mass moments (g/mol)	
Mn	1.146×10^5 (±1.632%)
Mp	1.528×10^5 (±0.797%)
Mv	n/a
Mw	2.702×10^5 (±0.713%)
Mz	6.120×10^5 (±1.768%)
Polydispersity	
Mw/Mn	2.357 (±1.781%)
Mz/Mn	5.338 (±2.406%)
rms radius moments (nm)	
Rn	42.6 (±4.5%)
Rw	59.6 (±1.6%)
Rz	86.9 (±0.7%)

Figure S2. Size-exclusion chromatogram report of commercial chitosan

(Batch 244/020208; degree of *N*-acetylation. DA < 1%, Mw = 270 kg/mol; Mn = 115 kg/mol, dispersity **Đ** = 2.3)

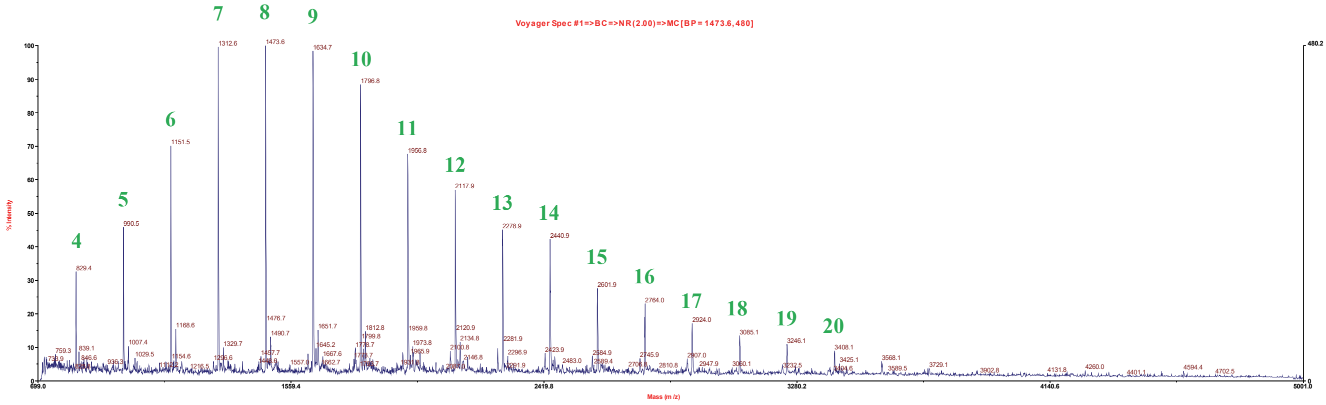

Figure S3. MALDI-TOF mass spectrum (positive linear mode) of COS-amf (D_{20}-M)

(Note that for each oligomer peak, the number of GlcN unit into the chain is given in green)

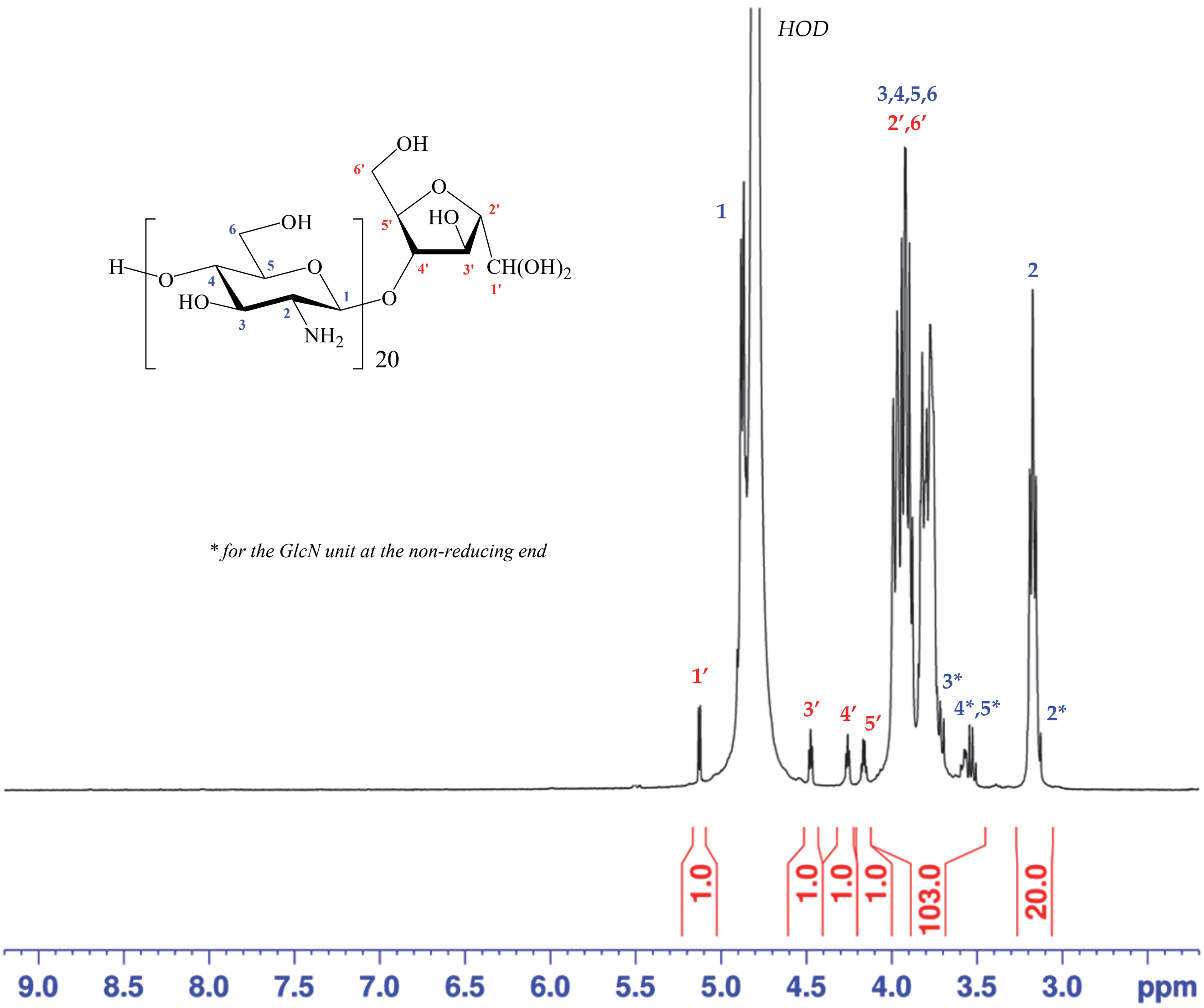

Figure S4. ^{1}H-NMR spectrum (D_2O, 500 MHz, 298 K) of COS-amf (D_{20}-M)

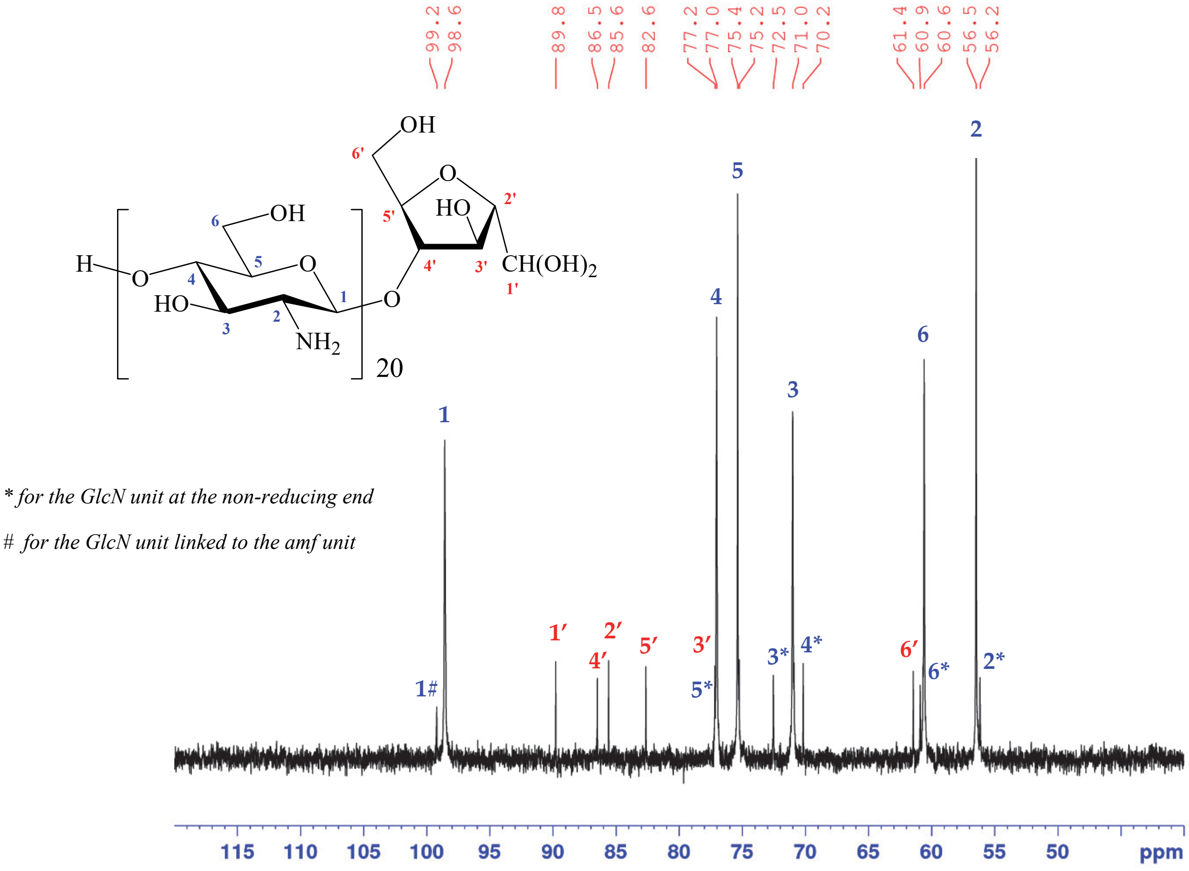

Figure S5. ^{13}C-NMR spectrum (D_2O, 125 MHz, 298 K) of COS-amf (D_{20}-M)

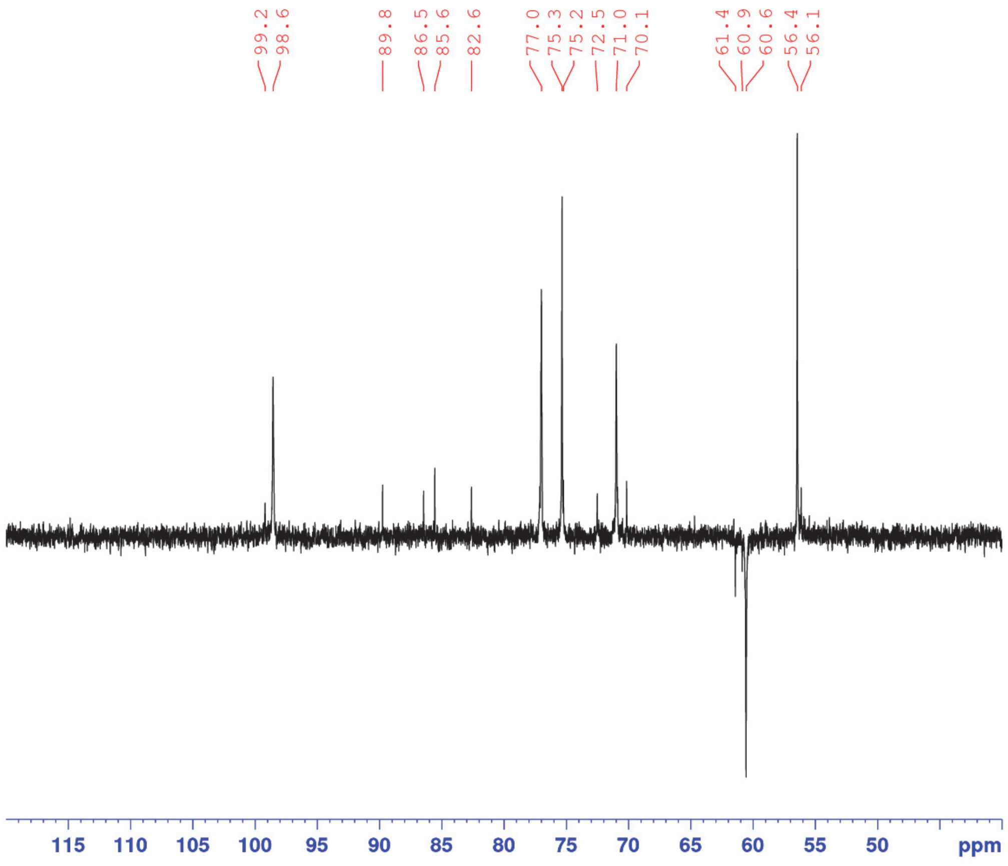

Figure S6. DEPT 135 ^{13}C-NMR spectrum (D_2O, 125 MHz, 298 K) of COS-amf (D_{20}-M)

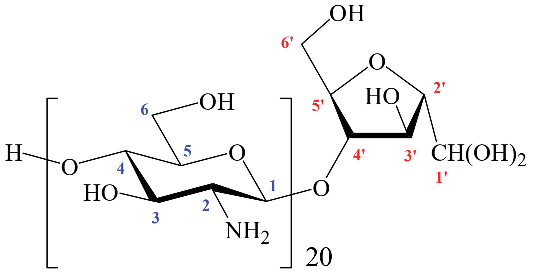

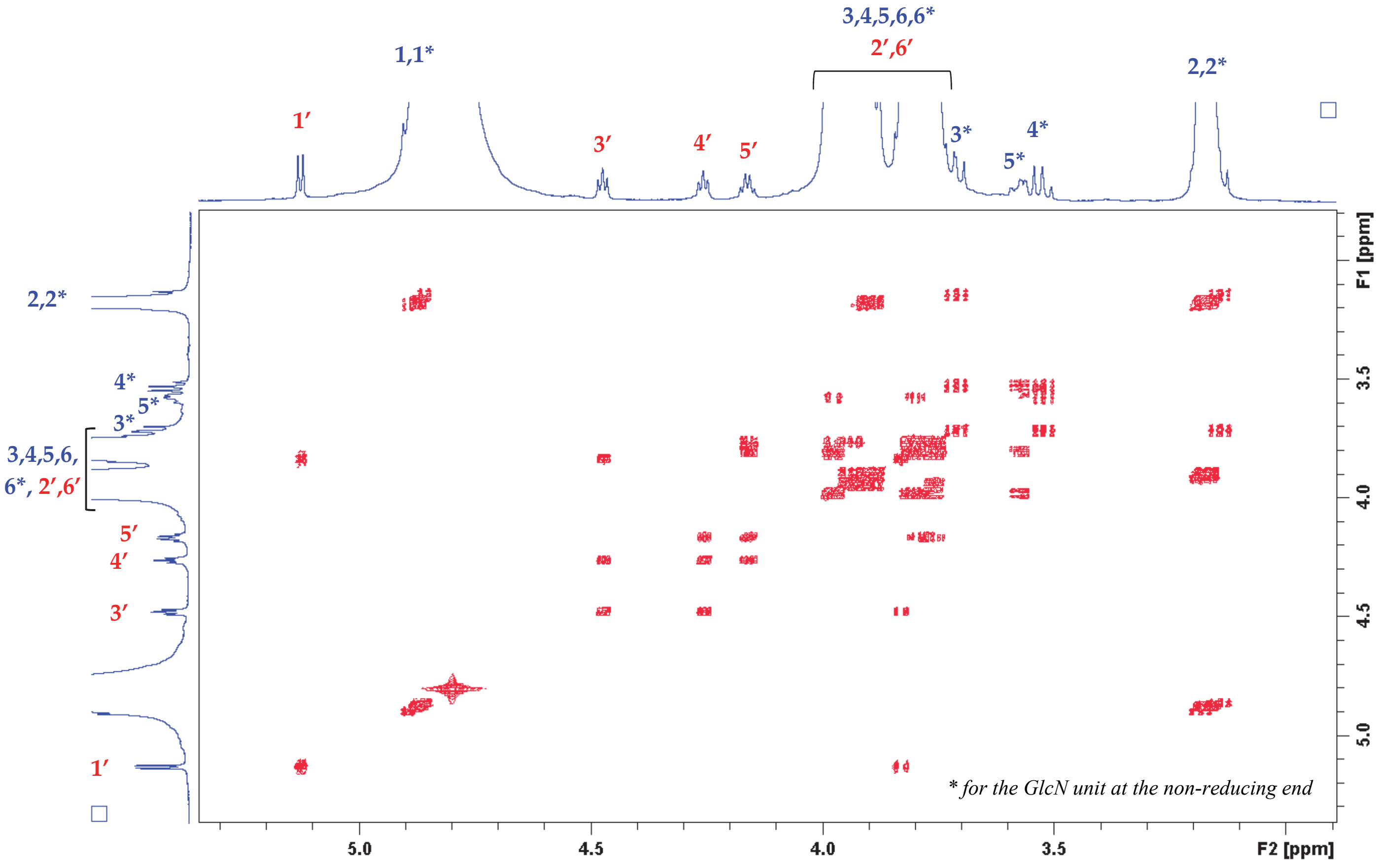

Figure S7. 2D COSY-NMR spectrum (D_2O, 500 MHz, 298 K) of COS-amf (D_{20}-M)

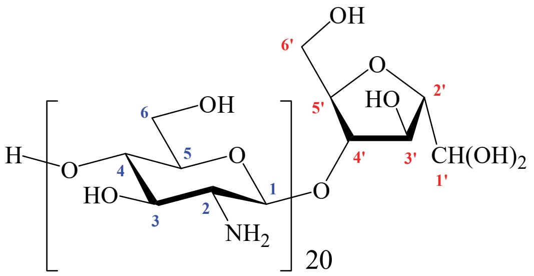

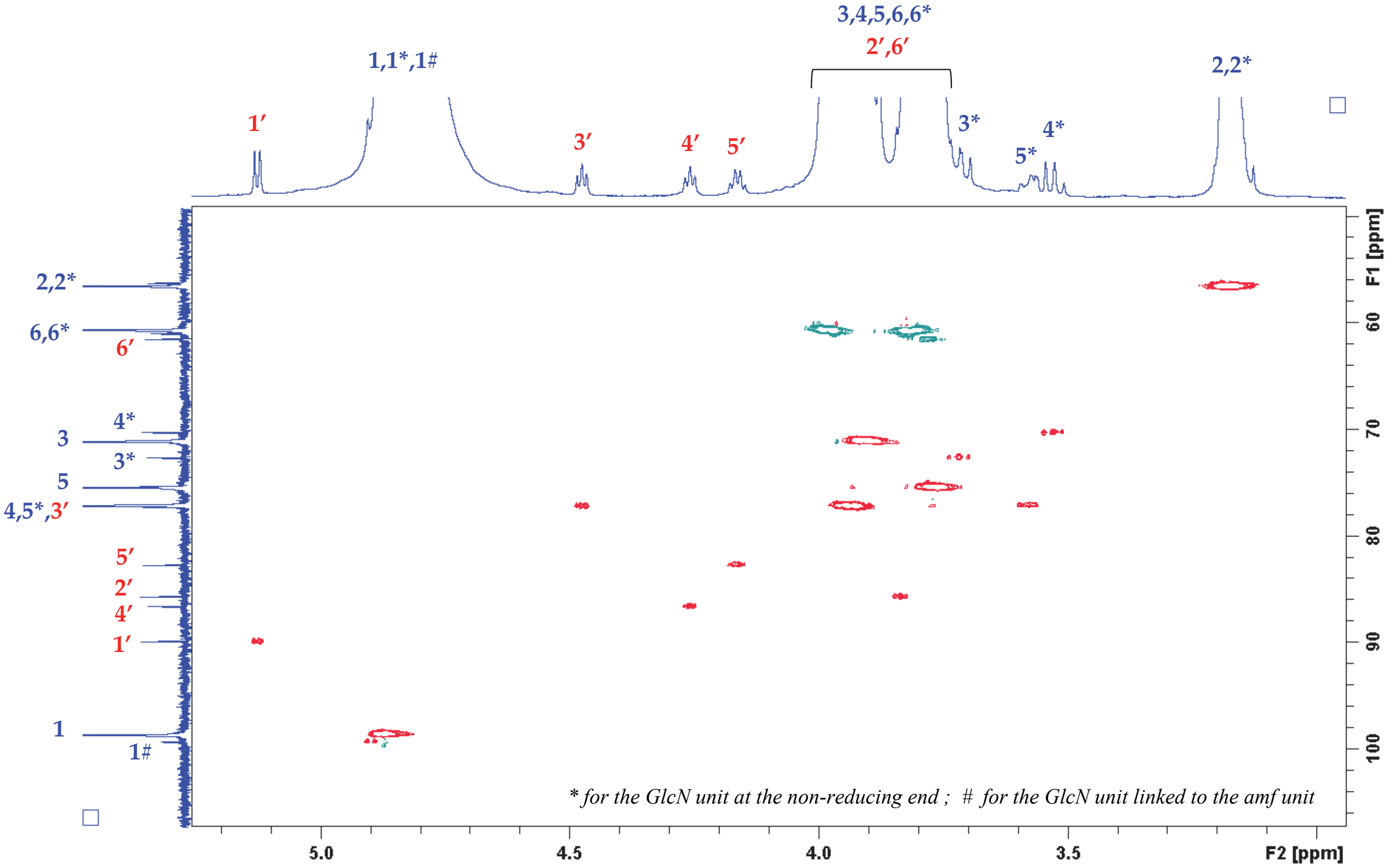

Figure S8. 2D HSQC-NMR spectrum (D2O, 500 MHz, 298 K) of COS-amf (D20-M)

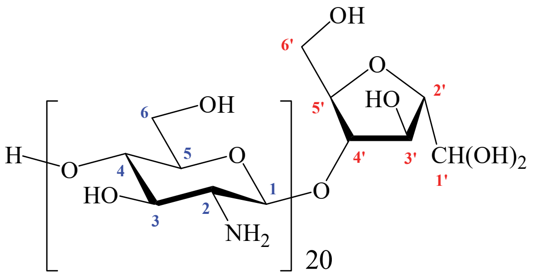

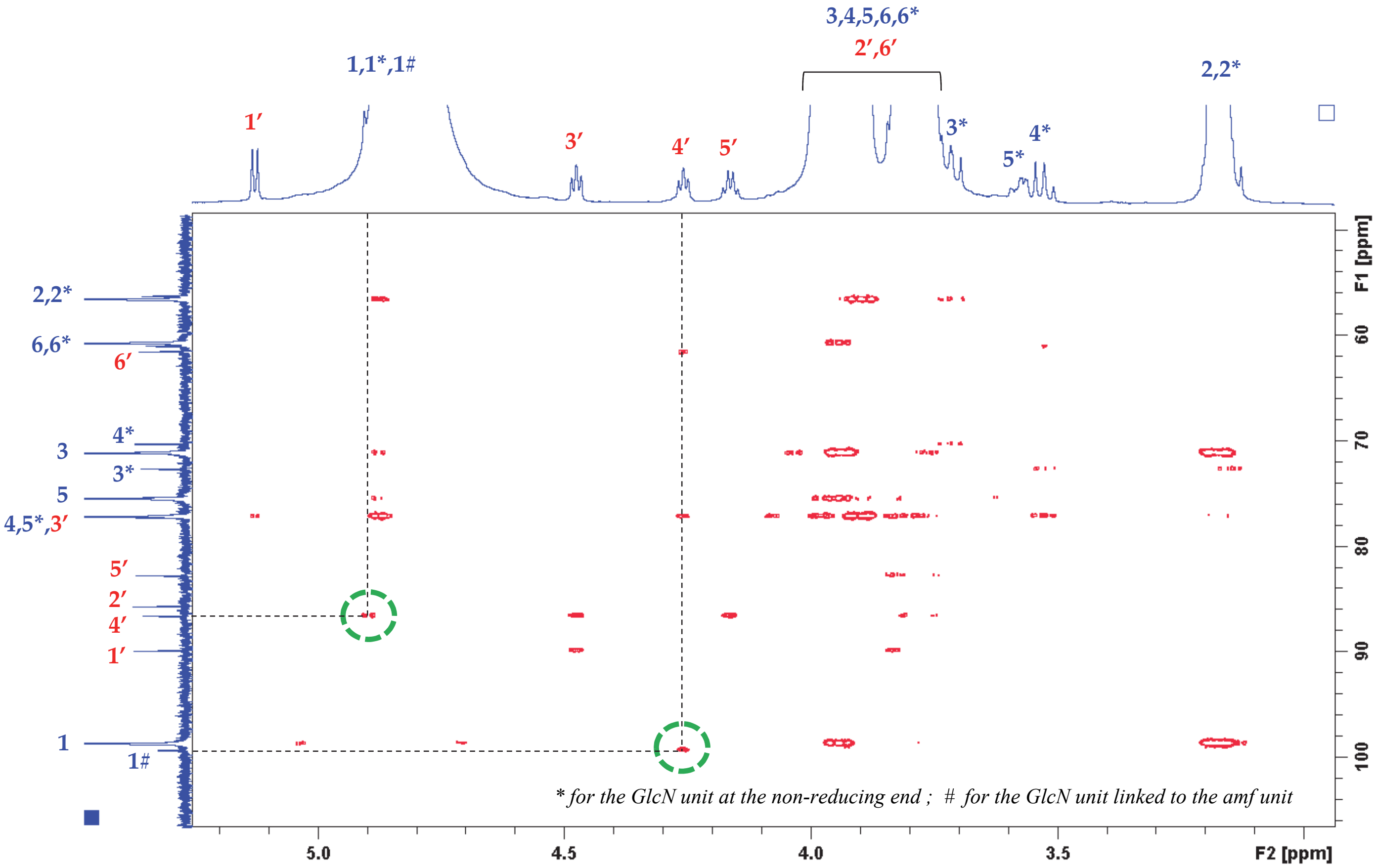

Figure S9. 2D HMBC-NMR spectrum (D_2O, 500 MHz, 298 K) of COS-amf (D_{20}-M)

(in green circles, ^1H-^{13}C correlations showing the connection between the amf unit and its neighbouring GlcN unit)

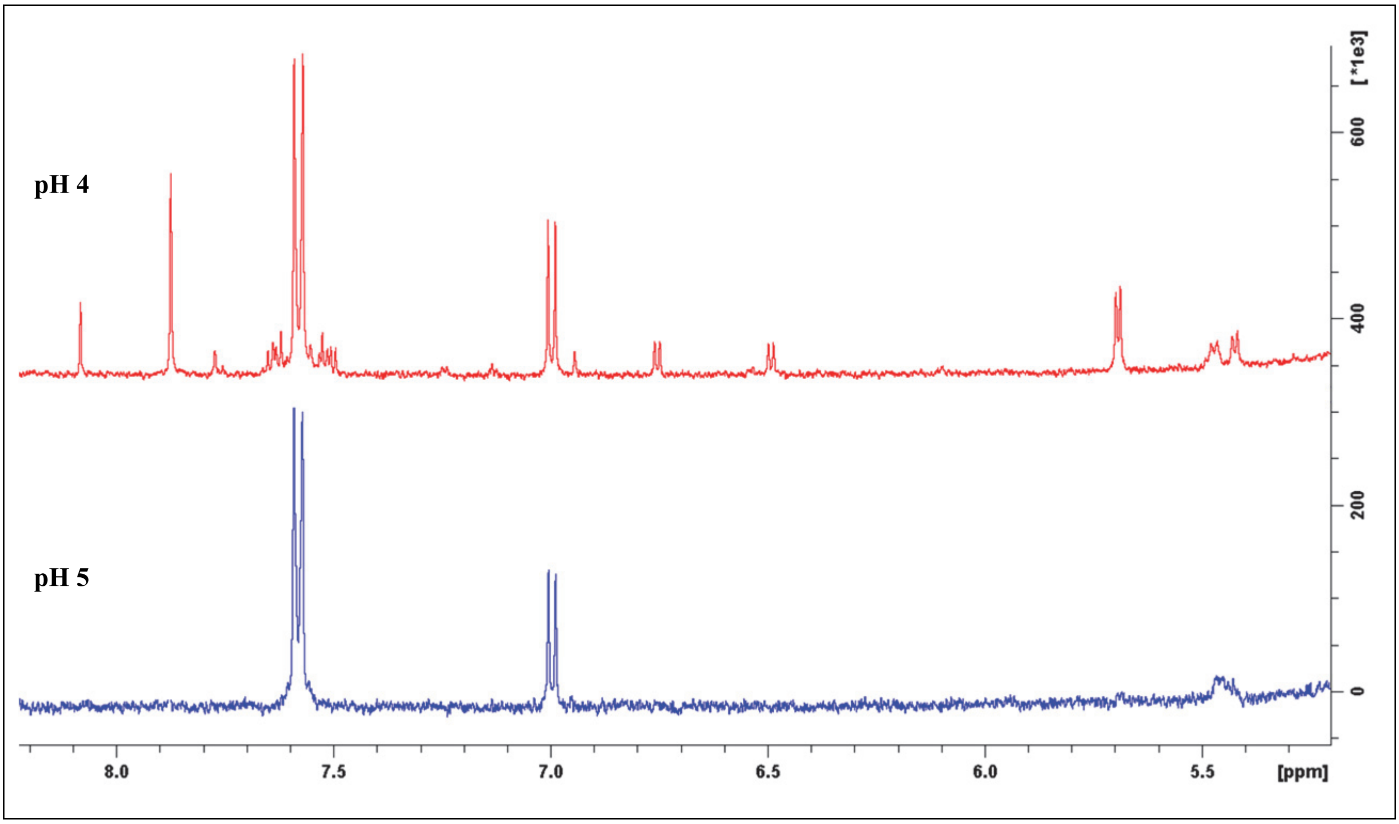

Figure S10. NMR spectra (region from 5.3 to 8.2 ppm) of the oximation of COS-amf (D_{20}-M) with PDHA at pH 4 and pH 5 after 1h of reaction

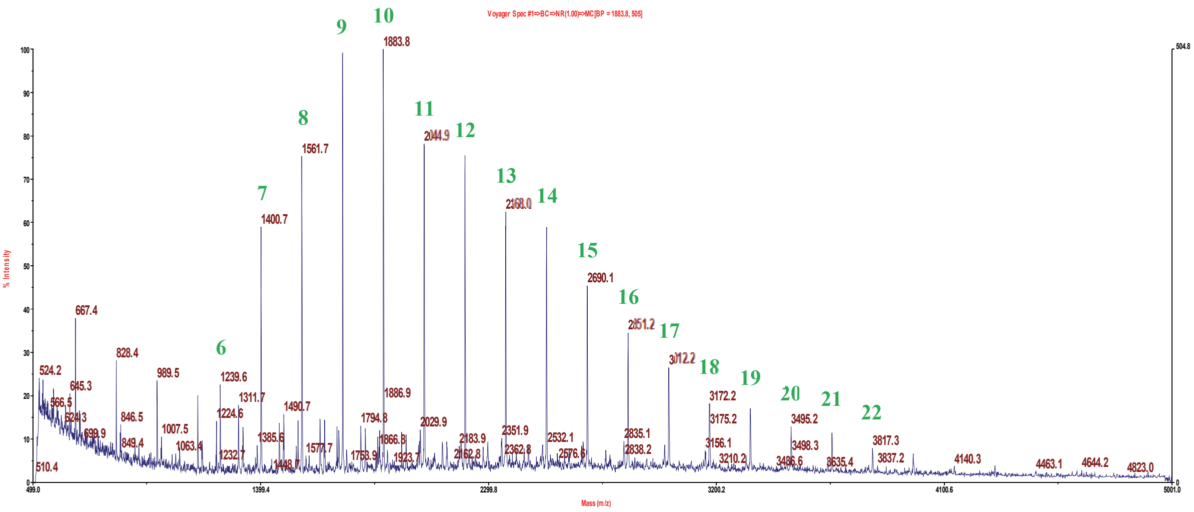

Figure S11. MALDI-TOF mass spectrum (positive reflectron mode) of conjugate **1**

(Note that for each oligomer peak, the number of GlcN unit into the chain is given in green)

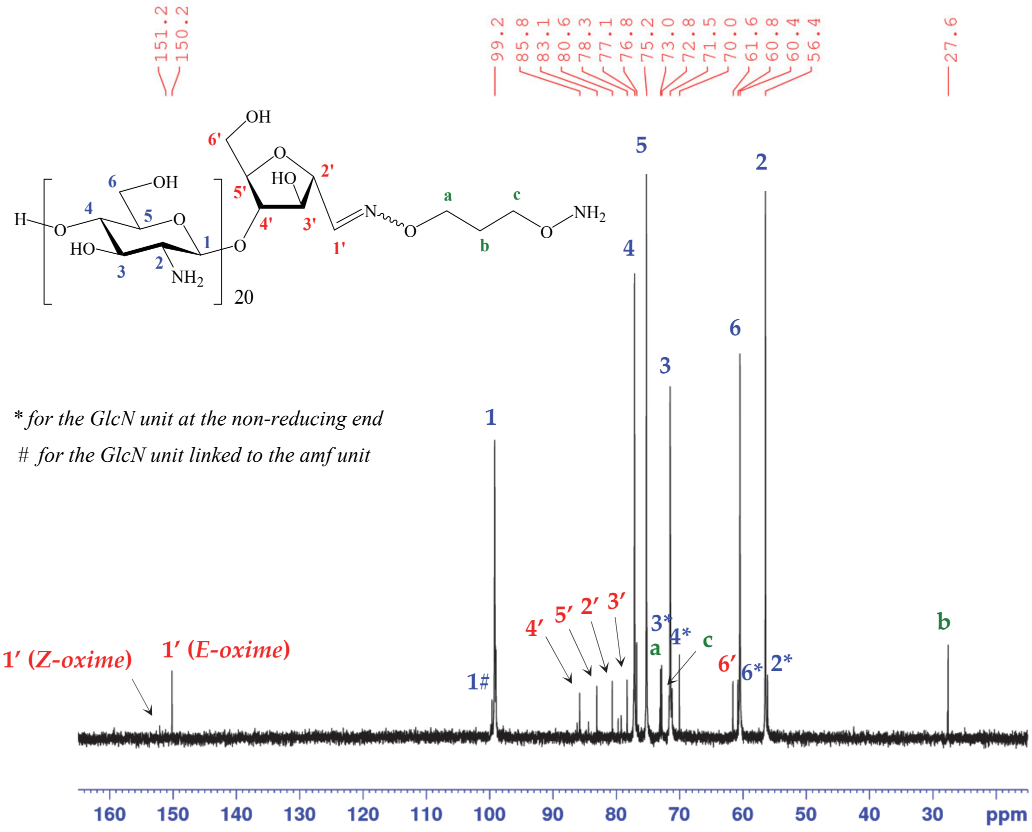

Figure S12. ^{13}C-NMR spectrum (D_2O, 125 MHz, 298 K) of conjugate **1**

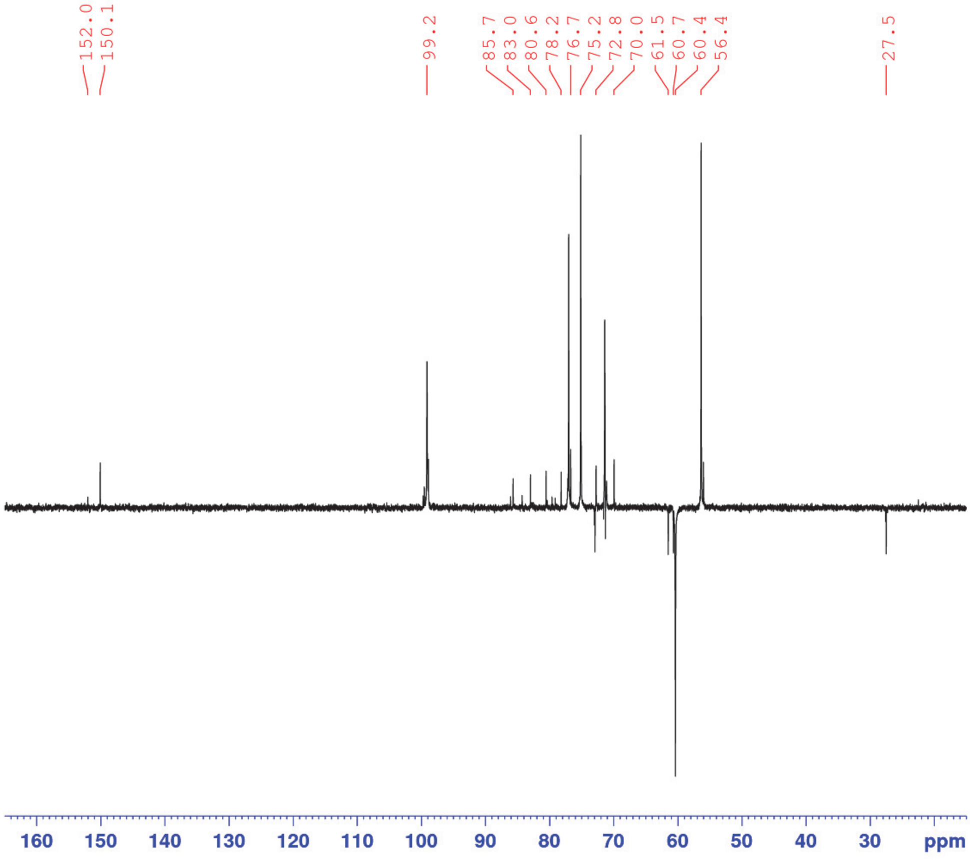

Figure S13. DEPT 135 ^{13}C-NMR spectrum (D_2O, 125 MHz, 298 K) of conjugate **1**

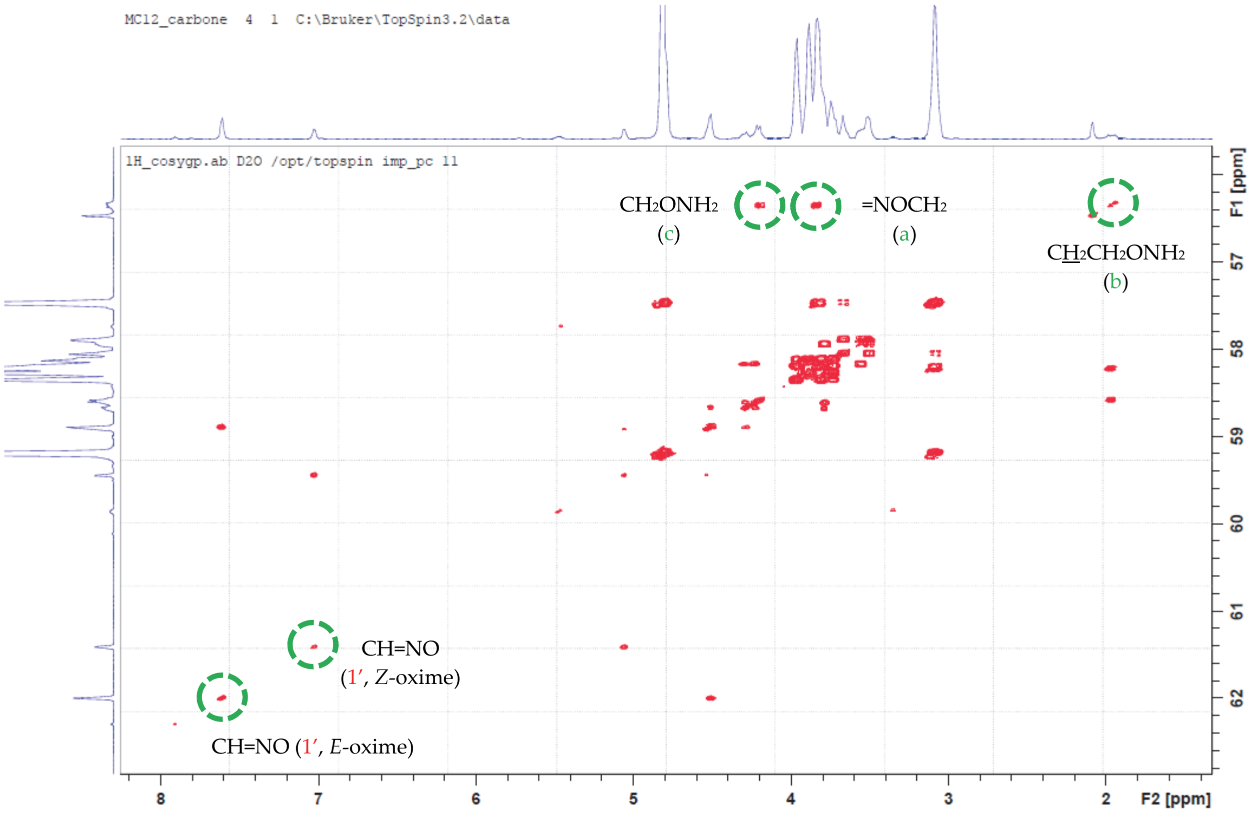

Figure S14. 2D COSY-NMR spectrum (D_2O, 500 MHz, 298 K) of conjugate **1**

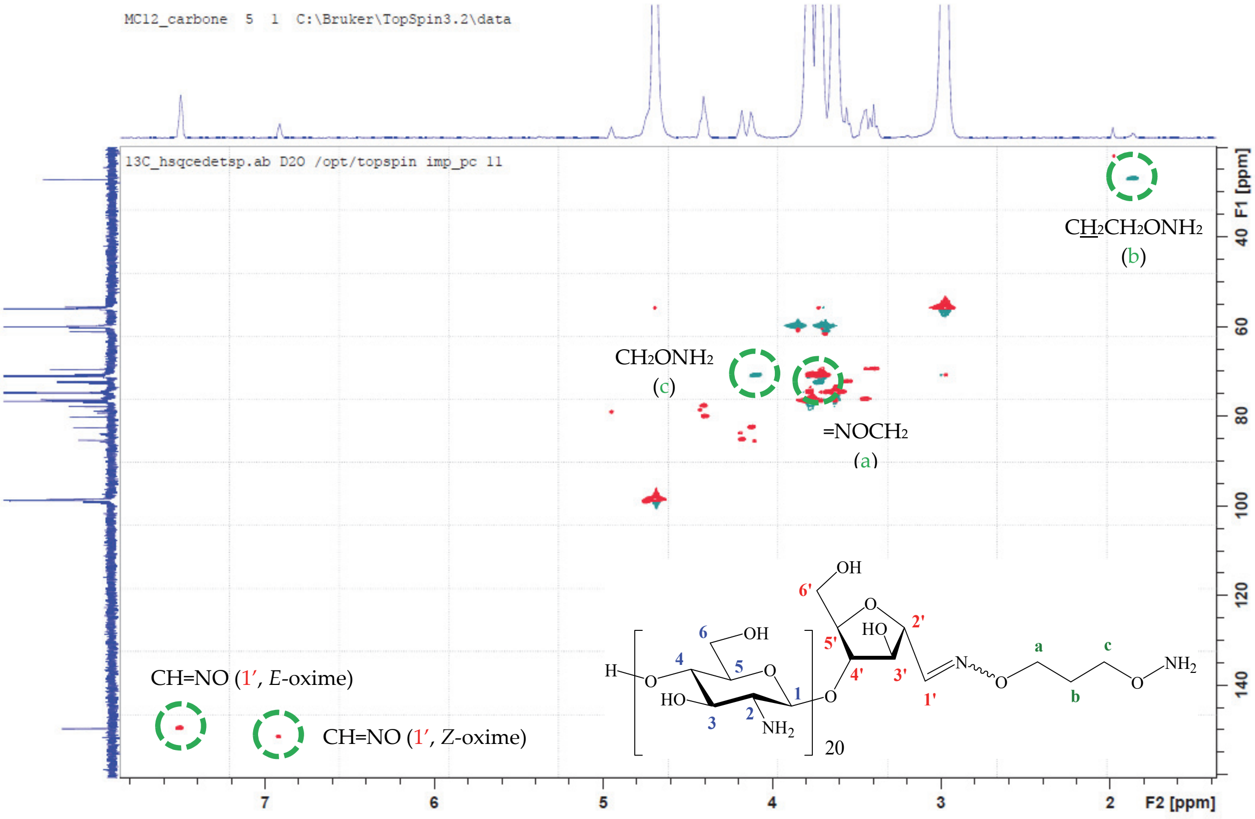

Figure S15. 2D HSQC-NMR spectrum (D_2O, 500 MHz, 298 K) of conjugate **1**

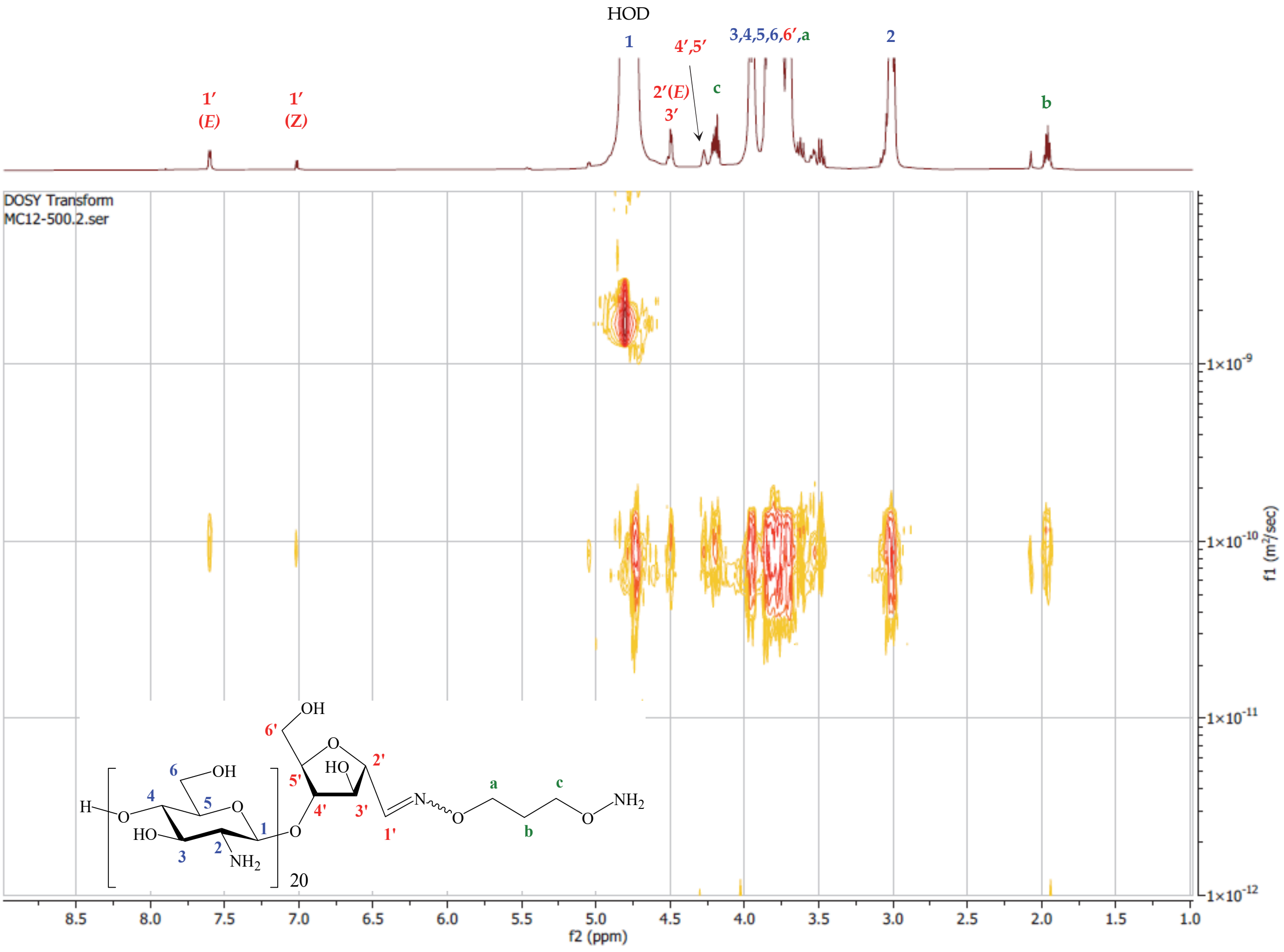

Figure S16. ^{1}H DOSY NMR spectrum (D_2O, 500 MHz, 298 K) of conjugate **1**

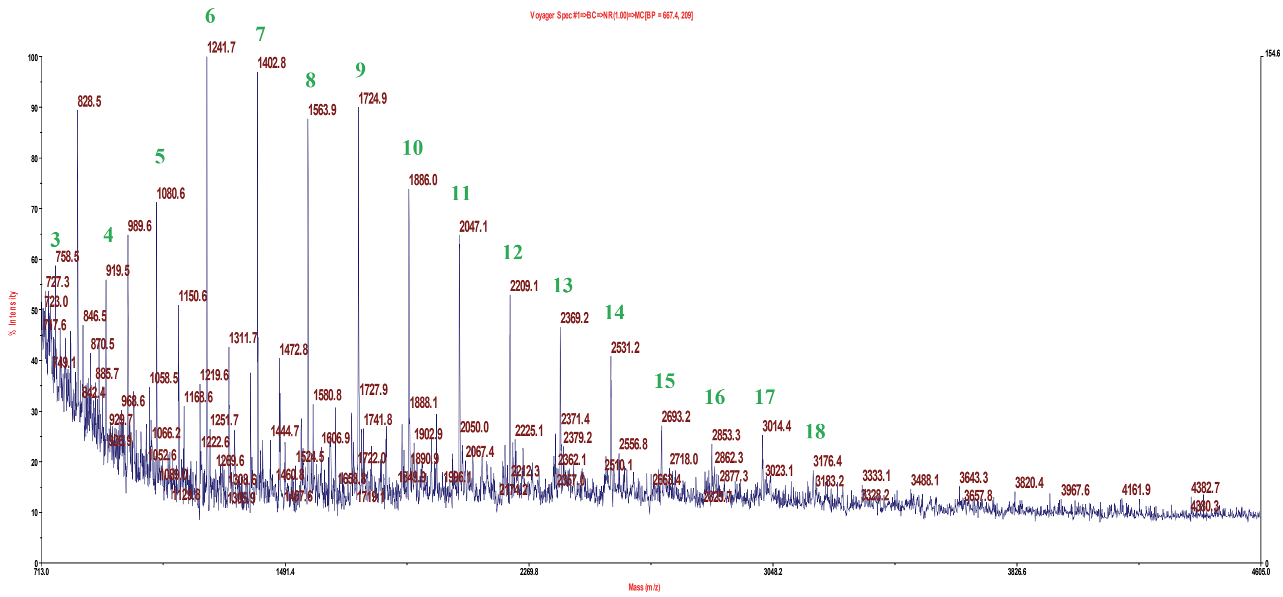

Figure S17. MALDI-TOF mass spectrum (positive reflectron mode) of conjugate **2**

(Note that for each oligomer peak, the number of GlcN unit into the chain is given in green)

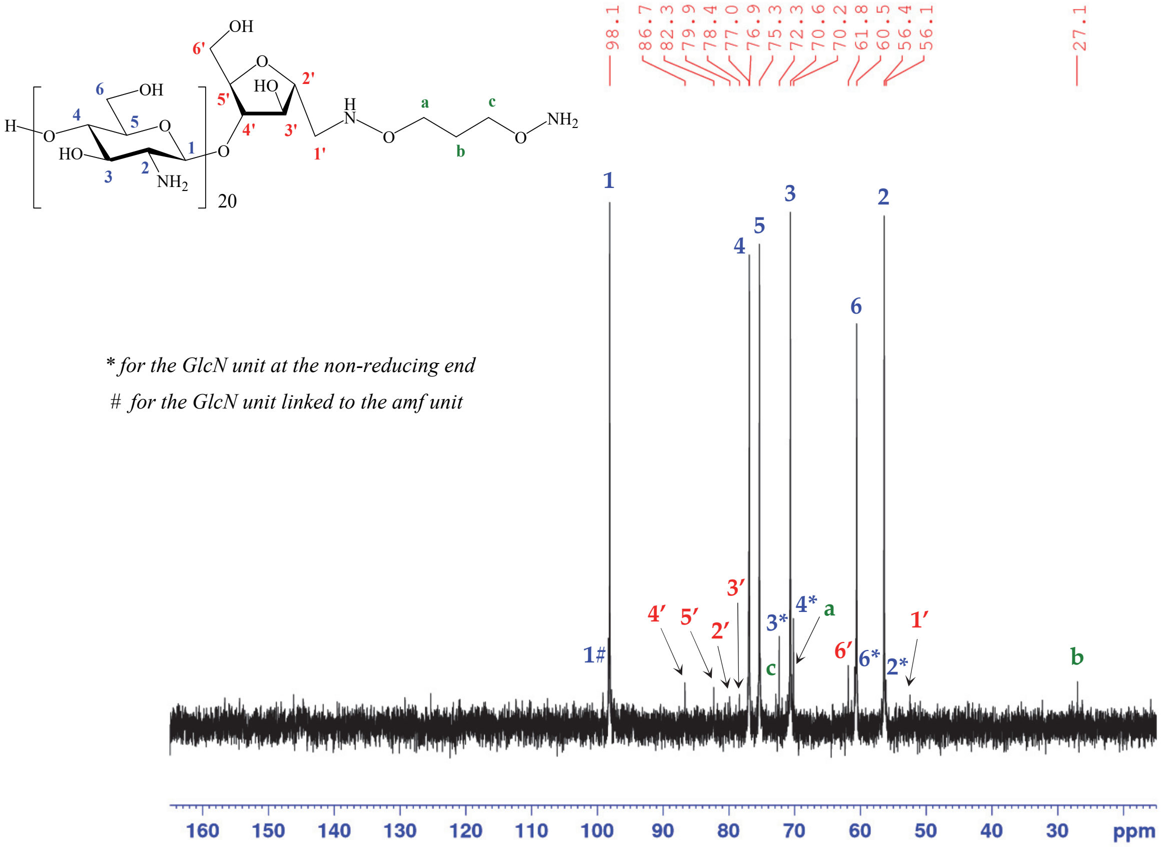

Figure S18. ^{13}C-NMR spectrum (D_2O, 125 MHz, 298 K) of conjugate **2**

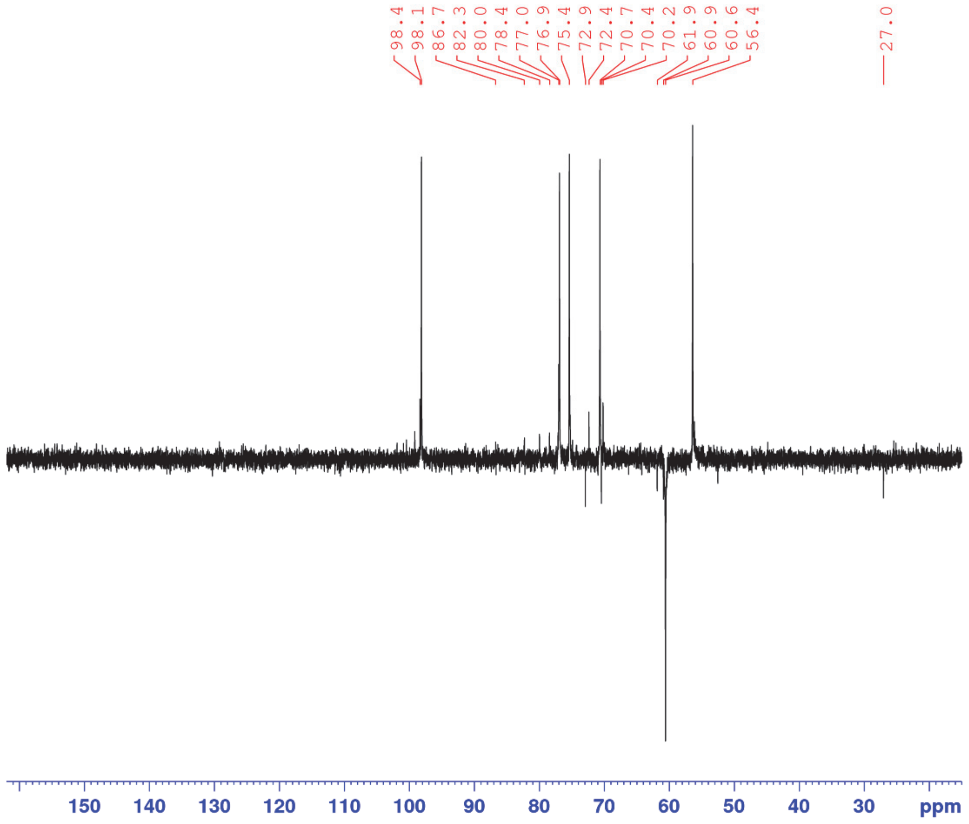

Figure S19. DEPT 135 ^{13}C-NMR spectrum (D_2O, 125 MHz, 298 K) of conjugate **2**

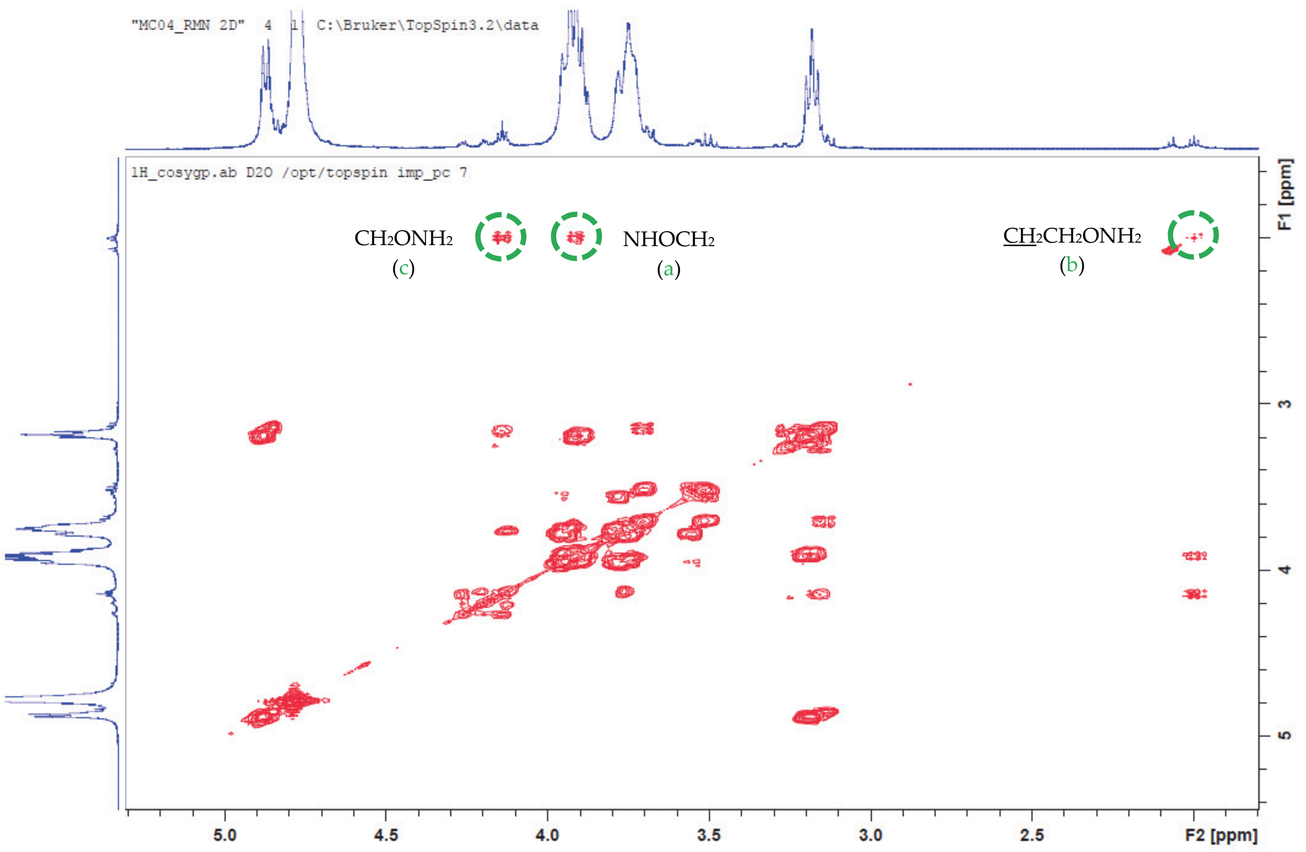

Figure S20. 2D COSY-NMR spectrum (D_2O, 500 MHz, 298 K) of conjugate **2**

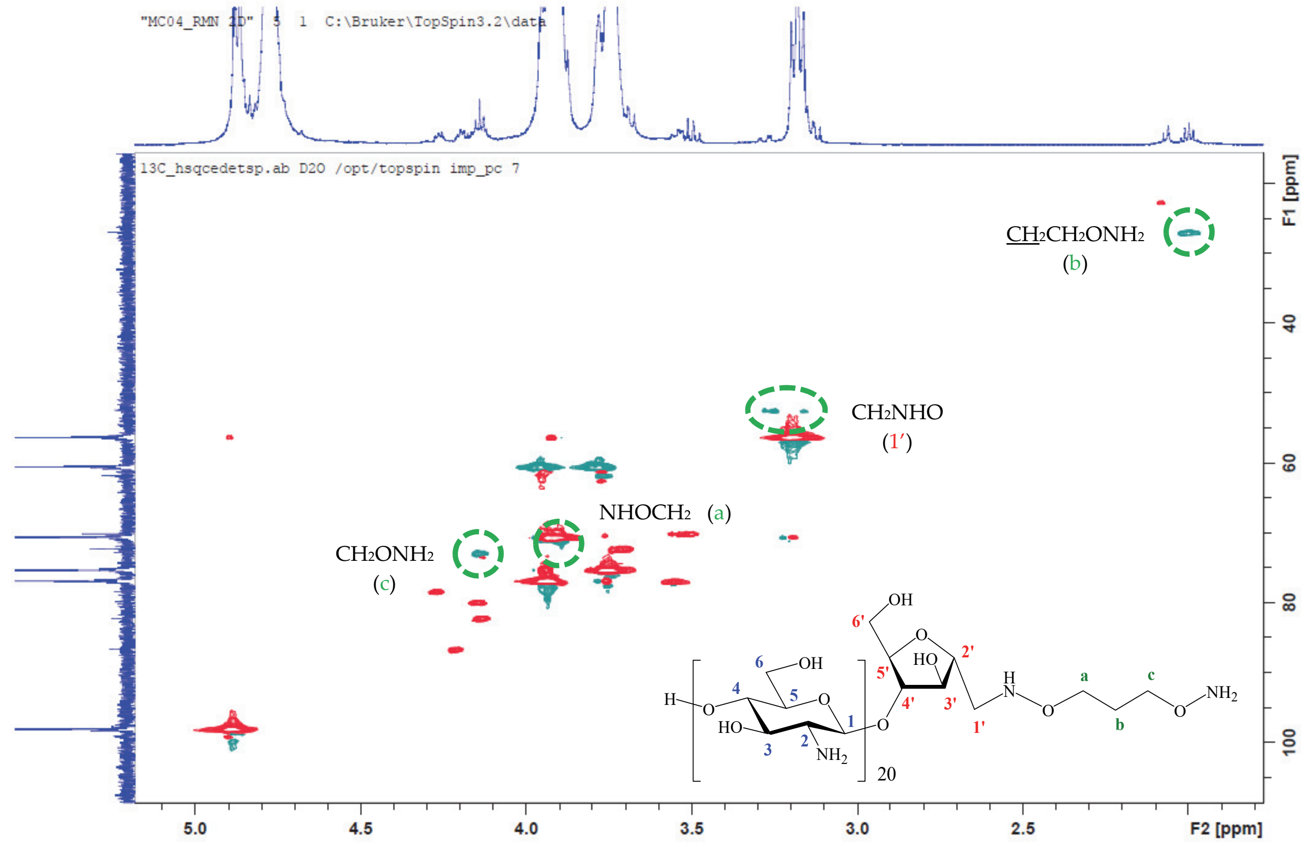

Figure S21. 2D HSQC-NMR spectrum (D_2O, 500 MHz, 298 K) of conjugate **2**

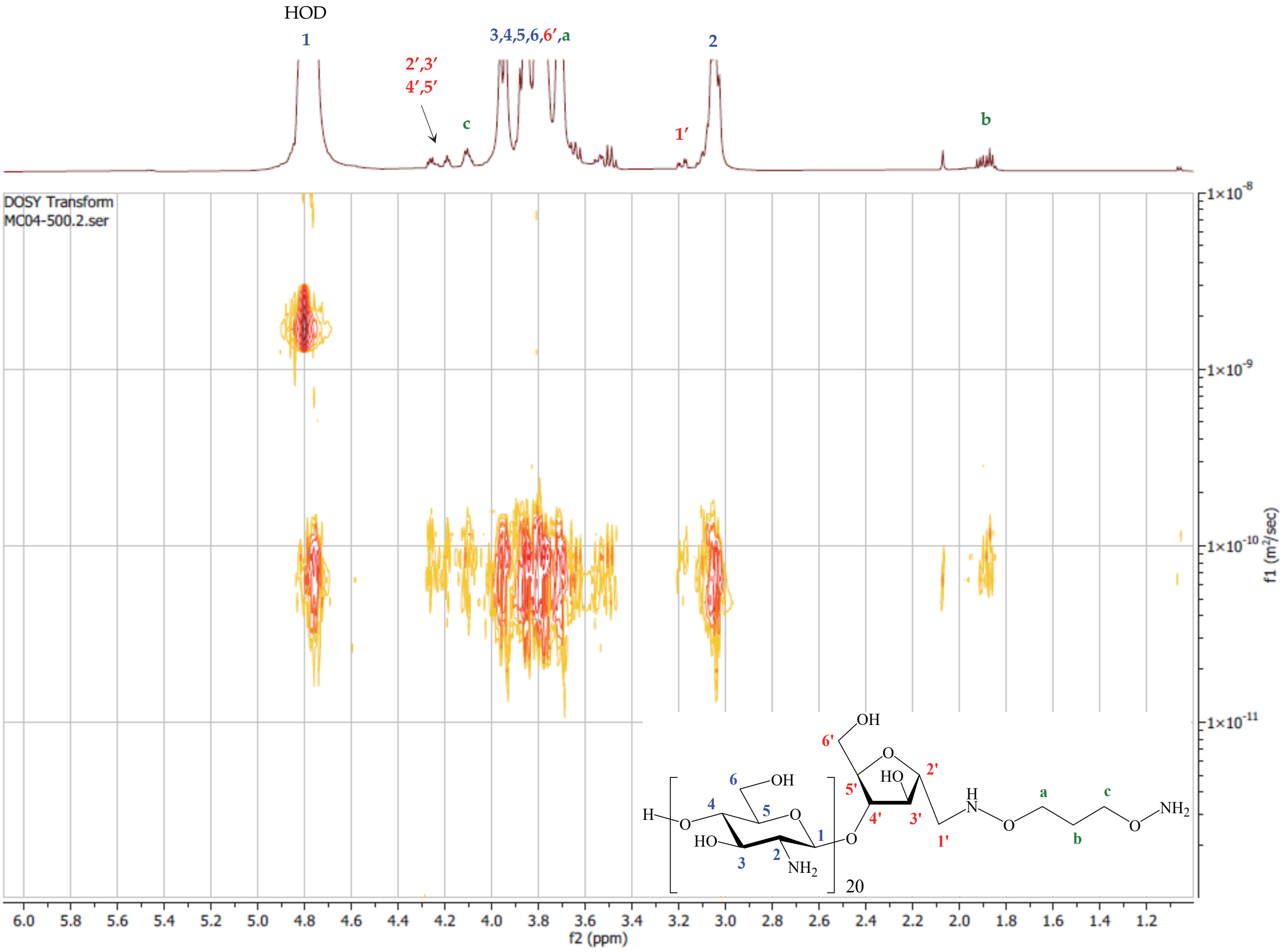

Figure S22. ^{1}H DOSY NMR spectrum (D_2O, 500 MHz, 298 K) of conjugate **2**
